# Hydrogels in the clinic

**DOI:** 10.1002/btm2.10158

**Published:** 2020-04-03

**Authors:** Abhirup Mandal, John R. Clegg, Aaron C. Anselmo, Samir Mitragotri

**Affiliations:** ^1^ John A. Paulson School of Engineering and Applied Sciences Harvard University Cambridge Massachusetts USA; ^2^ Wyss Institute for Biologically Inspired Engineering, Harvard University Cambridge Massachusetts USA; ^3^ Division of Pharmacoengineering and Molecular Pharmaceutics, Eshelman School of Pharmacy University of North Carolina at Chapel Hill Chapel Hill North Carolina USA

**Keywords:** clinics, drug delivery, FDA, injectable materials, marketed products, regenerative, translational medicine

## Abstract

Injectable hydrogels are one of the most widely investigated and versatile technologies for drug delivery and tissue engineering applications. Hydrogels’ versatility arises from their tunable structure, which has been enabled by considerable advances in fields such as materials engineering, polymer science, and chemistry. Advances in these fields continue to lead to invention of new polymers, new approaches to crosslink polymers, new strategies to fabricate hydrogels, and new applications arising from hydrogels for improving healthcare. Various hydrogel technologies have received regulatory approval for healthcare applications ranging from cancer treatment to aesthetic corrections to spinal fusion. Beyond these applications, hydrogels are being studied in clinical settings for tissue regeneration, incontinence, and other applications. Here, we analyze the current clinical landscape of injectable hydrogel technologies, including hydrogels that have been clinically approved or are currently being investigated in clinical settings. We summarize our analysis to highlight key clinical areas that hydrogels have found sustained success in and further discuss challenges that may limit their future clinical translation.

## 1 INTRODUCTION

Hydrogels are crosslinked hydrophilic polymer networks of high‐water content. Due to this high water content, hydrogels exhibit favorable biocompatibility and as such have been developed for and used in a variety of medical applications.^1^, ^2^, ^3^ Since hydrogel properties can be tuned through a variety of chemical approaches, their rational design and engineering has enabled new modalities for delivery of small molecules,^4^, ^5^, ^6^, ^7^ proteins,^8^, ^9^, ^10^, ^11^, ^12^ and cells^13^, ^14^ and as tissue engineering scaffolds for directing cell fate/lineage,^15^, ^16^ stem cell expansion, 17, 18, 19, 20 and tissue regeneration.^21^, ^22^, ^23^, ^24^, ^25^ Research efforts to develop hydrogels for biomedical applications represents one of the most studied areas at the interface of engineering and medicine.^26^ The translation of hydrogels into the clinic, especially for advanced hydrogel systems, remains a challenge despite many products and current clinical investigations. This article summarizes the present state of hydrogel materials in clinical medicine. Of all the hydrogels currently in preclinical development, studied in clinical trials, and used in the clinic as approved products, injectable hydrogels represent a subset that are capable of providing the most versatile deployment as delivery/regeneration platforms. Injectable hydrogels can be directly applied to sites of interest, independent of, and in many cases conforming to, local geometric/physiological constraints. For detail on the various scientific aspects of hydrogels, please refer to the following recent reviews.^26^, ^27^, ^28^, ^29^, ^30^, ^31^ In this review, we provide a general overview of the clinical landscape of injectable hydrogels, highlighting both approved hydrogel systems and current clinical trials. We conclude with a discussion of current challenges to bolster translation of hydrogel materials.

## 2 CLINICAL LANDSCAPE OF HYDROGELS

**FIGURE 1:**
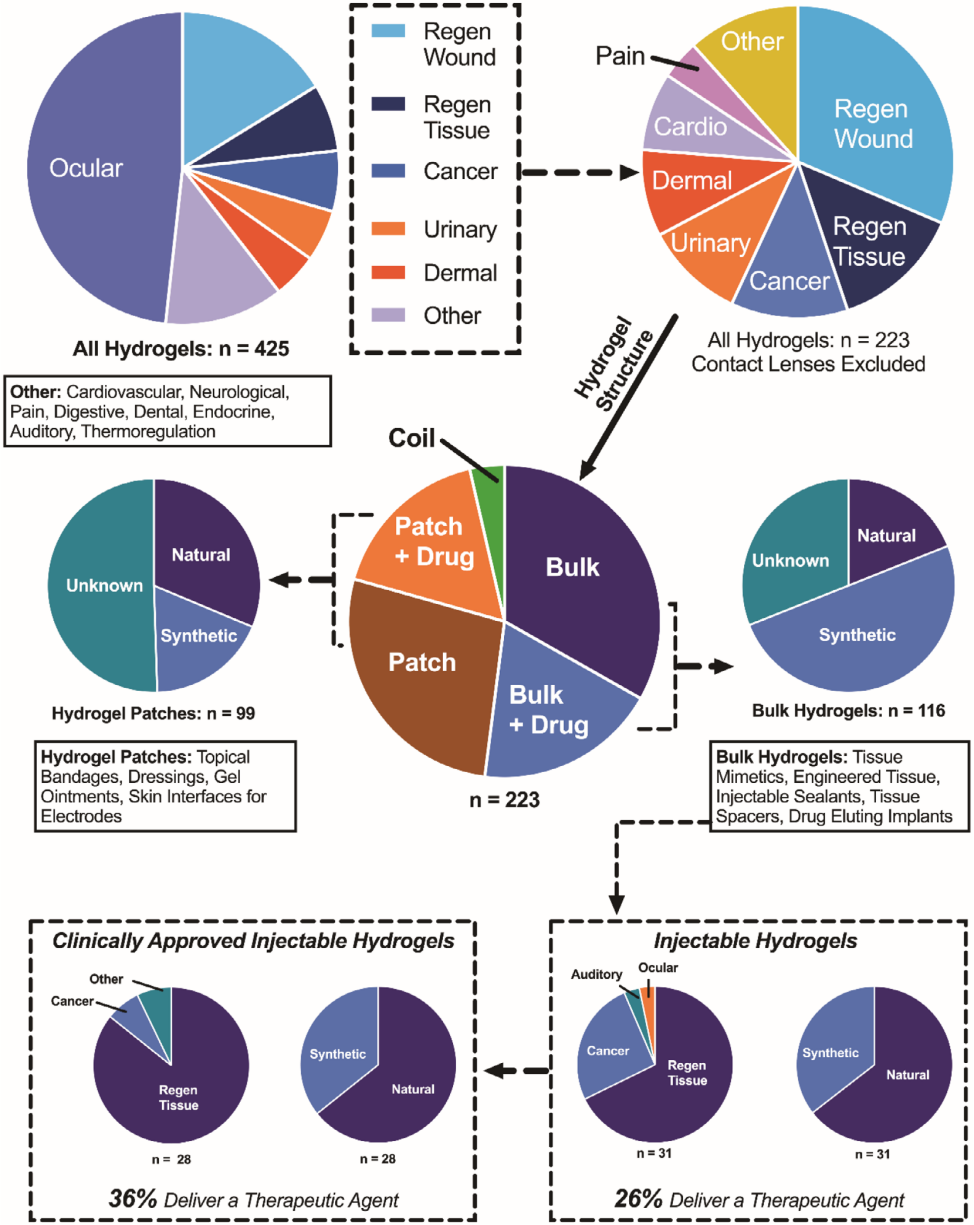
Comprehensive analysis of hydrogels in clinical trials. Clinical trials which mentioned a hydrogel were identified using http://clinicaltrials.gov. Trials where the hydrogel was not used for a diagnostic or therapeutic purpose were removed. Trials which investigated the hydrogel cleaning solution, or another aspect of the hydrogel packaging, were also removed. In total, there were 425 clinical trials involving hydrogel materials. The primary clinical application of hydrogel materials was for soft contact lenses (202 unique clinical trials). With contact lenses excluded from further analysis, there were 223 clinical trials, spanning diverse medical applications. Trials which used the hydrogel as a tissue substitute, or a mechanical support to augment existing tissues (Regen Tissue) were analyzed separately from those which used hydrogels as a dressing or barrier to facilitate healing of an abrasion, burn, or ulcer (Regen wound). Of the 223 non‐lens hydrogel clinical trials, 8 used a hydrogel coil (cardiovascular application), 99 used a hydrogel patch, and 116 used a bulk hydrogel. Of the 116 bulk hydrogels, 31 were delivered via injection. Within the domain of injectable hydrogels, there are 28 approved clinical products and 31 devices in clinical trial (for full detail, see Tables 1 and 2). Within each hydrogel grouping (i.e., patch, bulk, injectable), we also stratified clinical trials by material origin (i.e., natural, synthetic, or unknown). Material origin was determined from either the clinical trial description or the device's U.S. patent. Solid lines denote categorization or clarification of a group, while dotted lines represent extraction of a particular subset

As shown in Figure 1, the most abundant medical application of hydrogels is for soft contact lenses. As compared to glass lenses, hydrogels permit gas diffusion and retain water at the eye surface.^32^ Clinical trials on new hydrogel lens products have focused on a variety of outcomes, including extending the time of continuous wear, adding pigments, and optimizing the lens geometry. To better delineate the various applications and types of hydrogels in the clinic, we separated them based on their structure in terms of hydrogel patches, coils, or bulk materials. Within each structural grouping, hydrogels that partition and deliver a therapeutic agent were separated from hydrogels that exert their therapeutic function without delivering a drug. For the purpose of our analysis, we defined a hydrogel patch as any material which is topically applied. Hydrogel patches are mostly used to provide an engineered barrier between compromised tissue and the external environment. For example, in the treatment of burn wounds, a hydrogel patch can achieve any combination of (a) preventing bacterial growth within the wound, (b) delivering therapeutics which accelerate healing, and (c) maintaining a moist tissue environment thereby reducing pain. Specific hydrogel patches are currently being explored for facilitating healing processes in diabetic ulcers, treating skin conditions such as eczema and psoriasis, and more. While accounting for only eight of the hydrogel clinical trials, hydrogel coils are useful as a replacement for platinum coils in the treatment of aneurism. For these applications, hydrogels of natural (i.e., gelatin) or synthetic (i.e., acrylamide, acrylic acid, glycolic acid) materials have been utilized, as well as composite structures (i.e., hydrogel‐coated metal coils). The third and most abundant class of hydrogels in the clinic, bulk hydrogels, is typically used for tissue augmentation or regeneration. Bulk hydrogels can act as a filler or replacement for soft tissue (e.g., to treat osteoarthritis, lipoatrophy), or provide mechanical support for compromised tissue (e.g., urinary incontinence, myocardial infarction). Current efforts include fabricating cell‐laden bulk hydrogels for tissue regeneration (e.g., kidney augmentation, myocardial regeneration). Similar to hydrogel patches, bulk hydrogels can themselves exert a therapeutic action or act as a depot for delivering a bioactive agent.

We also break down each hydrogel conformation (i.e., bulk, patch) by their material origin. Hydrogel biomaterials are either natural or synthetic in material origin. Natural hydrogels are typically derived from polypeptides (e.g., fibrin, collagen, gelatin) or polysaccharides (e.g., hyaluronic acid, alginate, cellulose, chitosan).^33^ These natural polymers can gel through physical interactions between polymer chains, or in some cases by ionic crosslinking via multivalent ions. A major advantage of natural hydrogels, particularly as injectable or implantable materials, is that they are generally biocompatible and biodegradable. Synthetic hydrogels (e.g., poly(ethylene glycol), poly(acrylate) derivatives, poly(methacrylate) derivatives, poly(acrylamide), poly(lactic‐co‐glycolic acid), poly(vinyl alcohol), and poly(urethane)) afford high tunability during synthesis. This is because synthetic polymers’ molecular weights and crosslinking ratios can be controlled to tune hydrogel physical properties (i.e., swelling in water, viscosity, elasticity, porosity,). As such, material choice is one of the most important considerations for hydrogels, since material choice governs biocompatibility, porosity, degradability, and release of therapeutic agents. These material parameters may also be affected by the local physiological environment in which the hydrogel will be deployed.

Finally, we highlight injectable hydrogels, a highly investigated subset of hydrogels that can provide unique advantages due to their injection‐enabling properties. Injectable hydrogels can be delivered to various sites of the body with minimal discomfort while conforming to the site of application, thereby providing a versatile platform for applications in drug delivery and tissue engineering. Of all the clinical trials involving bulk hydrogels, injectable hydrogels make up 26%. For the purpose of this article, we focus on the clinical application and translation of injectable hydrogels.

## 3 CLINICALLY APPROVED HYDROGEL PRODUCTS

Currently there are over 30 injectable hydrogel‐based products that have been approved by the FDA and/or EMA **(**Table 1 **)**. Perhaps the most well‐known examples are commercially successes such Medtronic's INFUSE® (approx. $750 million) and Endo's Vantas® (approx. $20 million). Here, we discuss these currently approved injectable hydrogel products.

**TABLE 1:** Clinically approved injectable hydrogels, grouped by their material class and broad indication

Name (company)	Hydrogel material/payload (gelation mechanism)	Injection type	Approved indication	Approval (year)
Cancer: synthetic				
SpaceOAR® Hydrogel (Augmenix, Inc.)	Polyethylene glycol (chemical reaction)	Percutaneous	For protecting vulnerable tissues during prostate cancer radiotherapy	EMA (2010) FDA (2015)
Vantas® (Endo Pharmaceuticals)	Histrelin acetate, poly(2‐hydroxyethyl methacrylate), poly(2‐hydroxypropyl methacrylate) and gonadotropin releasing hormone (chemical reaction)	Subcutaneous	Palliative treatment of prostate cancer	FDA (2004) EMA (2005)
Facial correction: synthetic				
Radiesse® (+) (Merz Pharmaceuticals)	Hydroxylapatite, carboxymethylcellulose with Lidocaine (physical interaction)	Dermis	Correction of wrinkles and folds, stimulation of natural collagen production	FDA (2015)
Radiesse® (Bioform Medical, Inc.)	Hydroxylapatite, carboxymethylcellulose (physical interaction)	Dermis	For correction of facial folds and wrinkles, signs of facial fat loss and volume loss	EMA (2004) FDA (2006 for first indication)
Artefill® (Suneva Medical, Inc.)	Polymethylmethacrylate beads, collagen and lidocaine (physical interaction)	Dermis	Facial wrinkles and folds	FDA (2006)
Sculptra® (Sanofi Aventis U.S.)	Poly‐L‐lactic acid (physical interaction)	Dermis	For correction of signs of facial fat loss, shallow to deep contour deficiencies and facial wrinkles	EMA (2000) FDA (2004 for first indication)
Facial correction: natural				
Belotero balance® (+) Lidocaine (Merz Pharmaceuticals)	Hyaluronic acid with lidocaine (chemical reaction)	Dermis	Moderate to severe facial wrinkles and folds	FDA (2019)
Revanesse® Versa+	Hyaluronic acid with lidocaine (chemical reaction)	Dermis	Moderate to severe facial wrinkles and creases	FDA (2018)
Teosyal® RHA (Teoxane SA)	Hyaluronic acid (chemical reaction)	Dermis	Facial wrinkles and folds	EMA (2015) FDA (2017)
Revanesse® Versa/Revanesse® Ultra (Prollenium Medical Technologies Inc.)	Hyaluronic acid (chemical reaction)	Dermis	Moderate to severe facial wrinkles and creases	FDA (2017)
Restylane® Lyft, Restylane® Refyne, Restylane® Defyne (Galderma Laboratories, L.P.) Restylane® Silk (Valeant Pharmaceuticals North America LLC/Medicis) Restylane® Injectable Gel (Medicis Aesthetics Holdings, Inc.)	Hyaluronic acid with Lidocaine (chemical reaction)	Subcutaneous, dermis, lips	For correction of volume deficit, facial folds and wrinkles, midface contour deficiencies, and perioral rhytids	EMA (2010) FDA (2012 for first indication)
Belotero balance® (Merz Pharmaceuticals)	Hyaluronic acid (chemical reaction)	Dermis	Moderate to severe facial wrinkles and folds	EMA (2004) FDA (2011)
Juvéderm® XC (Allergan, Inc.)	Hyaluronic acid with lidocaine (chemical reaction)	Facial tissue	Correction of facial wrinkles and folds	FDA (2010)
Evolence® Collagen Filler (Colbar Lifescience l)	Collagen (chemical reaction)	Dermis	Moderate to deep facial wrinkles and folds	EMA (2004) FDA (2008)
Elevess® (Anika Therapeutics)	Hyaluronic acid with lidocaine (chemical reaction)	Dermis	Moderate to severe facial wrinkles and folds	FDA (2006) EMA (2007)
Juvéderm®/Voluma XC/Ultra XC/Volbella XC/ Vollure XC (Allergan, Inc)	Hyaluronic acid (chemical reaction)	Facial tissue, cheek, lips	For correction of facial wrinkles and folds, volume loss, and lip augmentation.	EMA (2000) FDA (2006 for first indication)
Hylaform® (Hylan B gel), Captique Injectable Gel, Prevelle Silk (Genzyme Biosurgery)	Modified hyaluronic acid derived from a bird (avian) source (chemical reaction)	Dermis	Correction of moderate to severe facial wrinkles and folds	EMA (1995) FDA (2004)
Collagen Implant, CosmoDerm® 1 human‐based collagen, CosmoDerm® 2 human‐based collagen CosmoPlast® human‐based collagen (Inamed Corporation/Allergan, Inc.)	Human collagen (CosmoDerm: physical interaction, CosmoPlast: chemical reaction)	Superficial papillary dermis	For correction of soft tissue contour deficiencies, such as wrinkles and acne scars	FDA & EMA (2003)
Fibrel® (Serono Laboratories)	Collagen (physical interaction)	Dermis	For correction of depressed cutaneous scars	FDA (1988)
Zyplast(R)® and Zyderm(R)® (Inamed Corporation/Allergan, Inc.)	Bovine collagen (chemical reaction)	Dermis	For correction of contour deficiencies	FDA and EMA (1981)
Spinal fusion: natural				
EUFLEXXA® (Ferring Pharmaceuticals Inc.)	Hyaluronic acid (physical interaction)	Intra‐articular	Knee osteoarthritis	FDA (2004) EMA (2005)
INFUSE® bone graft (Medtronic Sofamor Danek USA, Inc.)	Collagen and recombinant human bone morphogenetic protein‐2 (physical interaction)	Spinal injection	Spinal fusion, and spine, oral‐maxillofacial and orthopedic trauma surgeries	FDA (2002 for first indication)
Osteogenic protein 1(OP‐1®) implant, OP‐1® Putty (Stryker Biotech)	Collagen, carboxymethylcellulose, and recombinant OP‐1 (physical interaction)	Spinal injection	Posterolateral lumbar spinal fusion	FDA (2001)
Other: synthetic				
TraceIT® Hydrogel Tissue Marker (Augmenix, Inc.)	Polyethylene glycol (chemical reaction)	Percutaneous	Improved soft tissue alignment for image guided therapy	FDA (2013)
Supprelin LA® (Indevus Pharmaceuticals, Inc.)	Histrelin acetate, Poly(2‐hydroxyethyl methacrylate) (chemical reaction)	Subcutaneous	Central precocious puberty	EMA (2005) FDA (2007)
Bulkamid® hydrogel (Searchlight Pharma)	Polyacrylamide (chemical reaction)	Transurtheral	Female stress urinary incontinence	EMA (2003) FDA (2006)
Coaptite® (BioForm Medical, Inc.)	Calcium hydroxylapatite, sodium carboxymethylcellulose, glycerin (physical interaction)	Submucosal	Female stress urinary incontinence	EMA (2001) FDA (2005)
Other: natural				
Algisyl‐LVR® Hydrogel Implant (LoneStar Heart, Inc.)	Alginate (physical interaction)	Percutaneous	Advanced heart failure	EMA (2014)

### 3.1 Facial correction/aesthetic products

The improved biocompatibility especially of hyaluronic acid (HA)‐based hydrogels have found a wide application as temporary dermal fillers in soft tissue augmentation. Juvéderm®, one of the leading HA‐based products currently in the market is widely used for the correction of age‐related volume loss, moderate to severe facial wrinkles and folds and lip augmentation. These HA containing hydrogels, once injected beneath the skin surface, integrate into the dermal layer and attract and bind to water molecules, thus providing volume and fullness to the skin lasting for about 6–12 months. Some of these dermal fillers including Juvéderm XC®, Elevess®, Prevelle Silk®, Revanesse® Versa +, Restylane® contain lidocaine as an additional component to minimize the pain or discomfort associated with injection. Collagen is also commonly used and has the shortest lasting effect (3–4 months) among the injectable filler materials. Calcium hydroxylapatite and Poly‐L‐lactic acid are among the long‐lasting synthetic absorbable filler materials with effects lasting up to 1–2 years. Artefill®, the only nonabsorbable material (polymethylmethacrylate beads) product approved by the FDA to date, is a permanent synthetic injectable filler used for the treatment of nasolabial folds. The increased numbers of approved naturally derived hydrogels in comparison to synthetic ones as aesthetic products could be attributed to improved biocompatibility.

### 3.2 Cancer products

The hydrogel's ability to encapsulate and release small molecules and biologics have been used for synthetic depot systems with improved, sustained local therapeutic delivery. Endo's Vantas ® has been approved by the FDA as a subcutaneous hormonal therapy to prevent testosterone‐dependent prostate cancer cells to grow following testosterone release. Vantas® employs a cylindrically shaped diffusion‐controlled reservoir system to sustain the release of gonadotropin releasing hormone (GnRH) or luteinizing hormone releasing hormone (LH‐RH) over time. The implant contains Histrelin acetate, a nonapeptide analogue of GnRH with added potency compared to leuprolide acetate‐poly(lactic‐co‐glycolic acid) (PLGA)‐based nonhydrogel products (Lupron depot® and Eligard®). Collectively, these hydrogel depots provide considerable benefits in patient acceptance and convenience through providing long‐lasting benefits with a single injection. Augmenix's SpaceOAR® hydrogel is the latest of all to be approved for protecting patients undergoing radiation therapy for prostate cancer. Surprisingly, none of the natural hydrogels have been approved for use in cancer products, possibly due to the advantages in using synthetic gels as sustained release platforms.

### 3.3 Spinal fusion products

As scaffolding materials, hydrogels have garnered remarkable attention due to their ability to provide mechanical support to the existing natural tissues. Sustaining the functional transition of the scaffold during the healing process remains as one of the critical challenges in the hydrogel‐based product design in regenerative medicine. Stryker's, OP‐1® implant was one of the first products approved by the FDA (2001) to promote bone growth. However, later in 2004, OP‐1® surgical putty containing the OP‐1 protein and collagen was designated as a “humanitarian use device” for the treatment of rare conditions due to failing to demonstrate the efficacy of the device. Medtronic's autograft replacement therapy, Infuse® bone graft received FDA approval in 2002 due to its improved osteoinductivity. The product containing recombinant human bone morphogenetic protein‐2 (rhBMP‐2) together with the absorbable collagen sponge carrier (ACS) initiates the bone induction process once injected into the spine and eventually leads to bone remodeling and new blood vessels formation. Ferring's EUFLEXXA® was approved in 2004 for treating knee pain caused by osteoarthritis. Currently there are no synthetic hydrogel products that have been approved for use in regenerative market presumably owing to their low biocompatibility.

### 3.4 Other indications

Other than aesthetics, cancer and spinal fusion products, hydrogels have been approved in many other indications. LoneStar's Algisyl‐LVR® is the only hydrogel product derived from alginate that has been approved for advanced heart failure. While both Bulkamid® and Coaptite® have obtained approvals for the treatment of urinary incontinence in females, application of polyacrylamide‐based Bulkamid® for urinary disorders has witnessed a surge in the clinical trials. Additionally, TraceIT® hydrogel tissue marker was approved by the FDA for image‐guided soft tissue alignment in 2013 and has established this PEG‐based hydrogel system as one of the most explored synthetic hydrogel systems for imaging and spacing in cancer treatments in the clinical trials.

## 4 CURRENT HYDROGEL CLINICAL TRIALS

Considerable efforts in the clinic are focused on evaluating new injectable hydrogel‐based systems as therapeutic, diagnostic, and aesthetic agents. In this section, we will briefly review (a) the current landscape of hydrogel‐based systems currently being investigated in the clinic (Table 2 **)** and (b) the key technologies attempting to integrate advanced functions and improved biocompatibility into the hydrogel structures.

**TABLE 2:** Examples of current clinical trials for injectable hydrogels

Name (sponsor company/university)	Hydrogel material/payload (gelation mechanism)	Injection type	Indications	http://clinicaltrials.gov identifier (phase)
Tissue regeneration: synthetic				
Argiform (Research Centre BIOFORM)	Polyacrylamide/silver ions (chemical reaction)	Intra‐articular	Knee osteoarthritis	NCT03897686 (NA)
Aquamid (Henning Bliddal)	Polyacrylamide (chemical reaction)	Intra‐articular	Knee osteoarthritis	NCT03060421 (NA)
PAAG‐OA (Contura)	Polyacrylamide (chemical reaction)	Intra‐articular	Knee osteoarthritis	NCT04045431 (NA)
Aquamid (A2 Reumatologi Og Idrætsmedicin)	Polyacrylamide (chemical reaction)	Intra‐articular	Knee osteoarthritis	NCT03067090 (NA)
GelStix® Nucleus augmentation device (Dr med. Paolo Maino Viceprimario Anestesiologia)	Polyacrylonitrile (chemical reaction)	Intra‐discal	Degenerative disc disease	NCT02763956 (NA)
Tissue regeneration: natural				
Hymovis Viscoelastic Hydrogel (Fidia Farmaceutici s.p.a.)	High molecular weight hyaluronan (physical interaction)	Intra‐articular	Osteoarthritis	NCT01372475 (Ph III)
HYADD® 4 Hydrogel (Fidia Farmaceutici s.p.a.)	Non‐crosslinked hyaluronic acid alkylamide (physical interaction)	Intra‐articular	Knee osteoarthritis	NCT02187549 (NA)
Promedon	Hydroxyethyl cellulose (physical interaction)	Knee	Osteoarthritis	NCT04061733 (NA)
Algisyl‐LVR® device (LoneStar Heart, Inc.)	Alginate (physical interaction)	Intra‐myocardial	Heart failure and dilated cardiomyopathy	NCT01311791 (Ph II/III)
Algisyl device (LoneStar Heart, Inc.)	Alginate (physical interaction)	Intra‐myocardial	Moderate to severe heart failure	NCT03082508 (NA)
Neo‐kidney augment (inRegen)	Gelatin with selected renal cells (chemical reaction)	Kidney	Type 2 diabetes and chronic kidney disease	NCT02525263 (Ph II)
Renal autologous cell therapy (inRegen)	Gelatin with renal autologous cells (chemical reaction)	Renal cortex	Chronic kidney disease from congenital anomalies of kidney and urinary tract	NCT04115345 (Ph I)
The Second Affiliated Hospital of Chongqing Medical University	Unknown/human amniotic epithelial cells (mechanism unknown)	Uterine cavity	Asherman's syndrome	NCT03223454 (Ph I)
Naofumi Takehara	Gelatin with basic fibroblast growth factor (mechanism unknown)	Intra‐myocardial	Ischemic cardiomyopathy	NCT00981006 (Ph I)
VentriGel (Ventrix, Inc.)	Native myocardial extracellular matrix (physical interaction)	Trans‐endocardially	Myocardial infarction	NCT02305602 (Ph I)
Cancer applications: synthetic				
Absorbable Radiopaque Tissue Marker (Sidney Kimmel Comprehensive Cancer Center at Johns Hopkins)	Polyethylene glycol/TraceIT® (chemical reaction)	Between pancreas and duodenum	Imaging of pancreatic adenocarcinoma	NCT03307564
Memorial Sloan Kettering Cancer Center	Polyethylene glycol (chemical reaction)	Visceral pleura	Lung biopsy	NCT02224924 (Ph III)
Absorbable Radiopaque Tissue Marker (Washington University School of Medicine)	Polyethylene glycol/TraceIT® (chemical reaction)	Resection bed	Imaging of oropharyngeal cancer	NCT03713021 (Ph I)
Absorbable Radiopaque Hydrogel Spacer (Thomas Zilli, University Hospital, Geneva)	Polyethylene glycol/TraceIT® (chemical reaction)	Between the target (prostate/vagina) and the organ (rectum)	Spacing in radiation therapy for rectal cancer	NCT03258541 (NA)
Augmenix, Inc.	Polyethylene glycol/SpaceOAR® (chemical reaction)	Between the rectum and prostate	Spacing in radiation therapy for prostate cancer	NCT01538628 (Ph III)
Royal North Shore Hospital	Polyethylene glycol/SpaceOAR® (chemical reaction)	Between the rectum and prostate	Spacing in radiation therapy for prostate cancer	NCT02212548 (NA)
University of Washington	Polyethylene glycol/TraceIT® (chemical reaction)	Around circumference of the tumor bed	Imaging of bladder carcinoma	NCT03125226
Cancer applications: natural				
Gut Guarding Gel (National Cheng‐Kung University Hospital)	Sodium alginate/calcium lactate (physical interaction)	Submucosal	Gastroenterological tumor and polyps	NCT03321396 (NA)
Incontinence: synthetic				
Bulkamid (Karolinska Institutet)	Polyacrylamide (chemical reaction)	Transurethral	Midurethral sling surgery	NCT02776423
Bulkamid (Cantonal Hospital, Frauenfeld)	Polyacrylamide/botulinum toxin A (chemical reaction)	Intra‐vesical	Mixed urinary incontinence	NCT02815046 (NA)
Bulkamid (Contura)	Polyacrylamide (chemical reaction)	Transurethral	Stress urinary incontinence	NCT00629083 (NA)
Bulkamid (Helsinki University Central Hospital)	Polyacrylamide (chemical reaction)	Transurethral	Stress urinary incontinence	NCT02538991 (NA)
Bulkamid (Karolinska Institutet)	Polyacrylamide (chemical reaction)	Submucosal	Anal incontinence	NCT02550899 (Ph IV)
Other: synthetic				
Ocular Therapeutix, Inc.	Polyethylene glycol/OTX‐TKI (chemical reaction)	Intra‐vitreal	Neovascular age‐related macular degeneration	NCT03630315 (Ph I)
EUTROPHILL hydrogel (Assistance Publique ‐ Hôpitaux de Paris)	Polyacrylamide (chemical reaction)	Subcutaneous	HIV‐related facial lipoatrophy	NCT01077765 (Ph III)
Frequency Therapeutics	Poloxamer/FX‐322 (physical interaction)	Intra‐tympanic	Sensorineural hearing loss	NCT04120116 (Ph II)

### 4.1 Hydrogels in regenerative medicine

Since the first FDA approval of Infuse® bone graft over 18 years ago, surprisingly there are few injectable hydrogel products in the market for wound care, tissue engineering and regeneration. However, with the advancement in new material chemistries, polymer physics, fabrication capabilities, and understanding of tissue engineering, currently hydrogel scaffolds investigated in regenerative medicine comprises the largest number of clinical trials. Particularly, polyacrylamide‐based synthetic hydrogels are extensively explored for the treatment of knee osteoarthritis via intra‐articular injection. Among natural hydrogels, HA and hydroxyethyl cellulose are currently being investigated for the same indication. However, despite being highly effective and biodegradable, there are still adverse events such as inflammation, joint pain and joint effusion that limit the clinical translation of these synthetic matrixes.^34^ Additionally, apart from alginate, gelatin‐based hydrogels are widely investigated with cellular components for heart and kidney diseases due to improved interaction with natural biological matrices.

### 4.2 Hydrogel scaffolds in cancer care

It is not surprising that hydrogels are also being investigated for improved cancer care. In particular, synthetic hydrogels including TraceIT® and SpaceOAR® are being widely investigated for imaging of cancerous cells and protecting healthy cells from radiation induced damage. The TraceIT® hydrogel tissue marker consists of polyethylene glycol (PEG) hydrogel micro particles containing covalently bound iodine, which allows visualization of cancerous tissue up to 3 months using magnetic resonance imaging (MRI), computed tomography (CT), and/or ultrasound for future surgical procedures. While the SpaceOAR® hydrogel is primarily designed to protect normal tissues from radiation injury during radiation treatment of cancerous tissues. However, only one of the natural hydrogels is currently being investigated in the clinic for cancer therapy which could be attributed to their limited structural integrity in comparison to the synthetic hydrogels. Thus, improving rigidity and persistence of natural hydrogels might hold significant promise in cancer applications.

### 4.3 Hydrogels for urinary incontinence

Likewise, synthetic polyacrylamide‐based Bulkamid® hydrogel has dominated the investigations in the clinical trials and has already been approved in 25 countries including Europe. It is currently being investigated in multicenter clinical trials across 33 sites in the United States and Canada. Currently there are no clinical trials that are exploring natural injectable hydrogel therapies presumably due to their achieving suitable rigidity for use as a spacer for incontinence indications.

### 4.4 Hydrogels for other indications

Injectable hydrogels for ocular applications have remained one of the most investigated areas in the preclinical space,^35^ but surprisingly there are no approved products in the clinic. Ocular Therapeutix's OTX‐TKI (tyrosine kinase inhibitor microcrystals in PEG hydrogel) is the only candidate currently in the clinical trials for the treatment of age‐related macular degeneration (AMD) following intravitreal injection. Intra‐tympanically administered FX‐322, a combination of two proprietary, small‐molecule drugs in poloxamer hydrogel has able to grab fast track designation by the FDA following successful phase 1/2 trials for the treatment of sensorineural hearing loss. Additionally, while there are numerous natural hydrogel (e.g., HA‐based, collagen‐based) aesthetic products in the market, polyacrylamide‐based hydrogels are currently being investigated for facial lipoatrophy. The broad scope of the hydrogel‐based aesthetic products in clinical trials could warrant a separate, and more focused, review.

## 5 DESIGN CHALLENGES

A hydrogel must meet application‐specific design criteria to suitably treat a medical condition. Broadly, these design criteria can be defined as either physical, chemical, or biological. Here, we will discuss the current status of hydrogel design and fabrication, as it pertains to each design challenge.

### 5.1 Mechanical robustness

Injectable hydrogels must have a sufficiently low viscosity to be introduced via a needle and syringe and a sufficient elasticity in situ to maintain their injected volume and sustain repetitive load. Overcoming this paradox is a major design challenge. One approach is to use a shear thinning polymer, such as HA.^36^, ^37^ As described in the previous section HA is used currently as a dermal filler, and is being investigated as an injectable replacement for cartilage. These materials gel by physical mechanisms (i.e., intermolecular interactions between polymer chains) which are disrupted by the shear of injection.^27^ Other clinically approved hydrogels that form via a physical mechanism are hydroxyapatite‐carboxymethylcellulose and collagen.

Another approach is to use in situ crosslinking to inject a hydrogel precursor which gels via a chemical reaction either within a mixing tip or within the physiological environment.^38^, ^39^, ^40^ Depending upon the application of interest, these chemical crosslinking reagents can be biodegradable or nonbiodegradable. For example, the Bulkamid (polyacrylamide) hydrogel employs a nondegradable crosslinking agent, while the SpaceOAR and TraceIT (polyethylene glycol) hydrogels each employ a hydrolytically degradable crosslinker.

**FIGURE 2:**
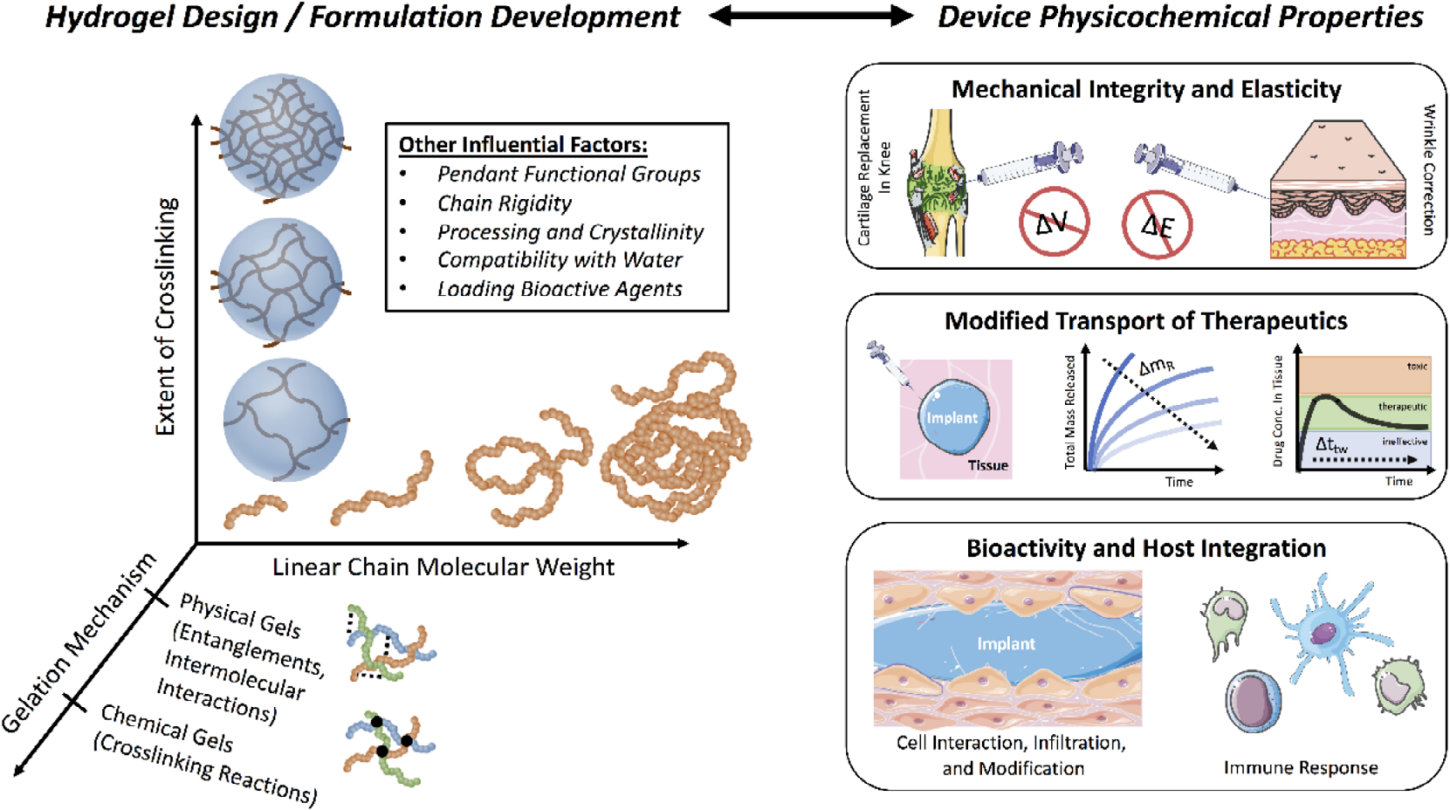
Design of hydrogels to overcome biophysical and biochemical challenges. When designing a new hydrogel, one determines the chemical functionality and chain rigidity by either selecting or synthesizing a proper backbone material (e.g., hyaluronic acid, polyethylene glycol, polyacrylate). The molecular weight of that linear backbone, the mechanism of crosslinking/gelation, as well as the molecular weight between crosslinks (i.e., extent of crosslinking) will determine the physical properties of the system. The combination of these chemical and physical identities will determine the gels’ mechanical integrity, solute transport properties, and interactions with host cells. Shown above are the clinical applications of (top) intra‐articular or subcutaneous injection, (middle) drug elution from an injected hydrogel depot, and (bottom) cell infiltration of an injected hydrogel scaffold

As illustrated in Figure 2, there are a number of hydrogel parameters that can be optimized to achieve specific mechanical properties. In particular, the molecular weight of polymer chains, extent of crosslinking, and crosslinking mechanism. In general, the viscosity of a polymer solution scales linearly with the molecular weight.^41^, ^42^ The hydrogel elastic modulus scales inversely with the molecular weight between crosslinks.^43^, ^44^ The crosslinking mechanism is determined by the chemical functionality of the polymer and any crosslinking agents.

### 5.2 Loading and release of therapeutic agents

Therapeutic agents, which can be delivered to the surrounding environment by an injectable hydrogel carrier, can include small molecules, macromolecules (i.e., peptides, proteins, nucleic acids), or engineered cells. Cargo release from injected hydrogels is determined by the (a) size of the cargo, relative to the mesh size of the hydrogel and (b) affinity of the cargo–gel interaction.

Injectable hydrogels currently used in the clinic that include a therapeutic agent deliver a small molecule drug or a biologic (microparticle depot systems). The most common therapeutic agent is lidocaine, an anesthetic drug that reduces the pain associated with the subcutaneous hydrogel injection. Lidocaine is included as a therapeutic agent within a number of hyaluronic acid hydrogels approved for facial correction applications. As a small molecule, lidocaine's release from the injected hydrogel is minimally perturbed by the hydrogel mesh. Lidocaine's release is likely quite rapid from these gels, similar to what has been seen in the published literature.^45^ For other drug‐eluting hydrogel applications, such as active wound dressings, a sustained release of protein drugs is needed. In these cases, one must either reduce the hydrogel mesh size via crosslinking, to perturb solute elution, or increase the hydrogel‐drug affinity with tailored gel compositions to increase retention.

### 5.3 Hydrogel bioactivity

For advanced tissue regeneration purposes, host cells must infiltrate, modify, and degrade bulk hydrogel materials. To achieve this aim, cells must adhere to the material. Some natural polymers, such as fibrin,^46^ collagen, gelatin,^47^ and HA,^40^ are naturally adhesive.^48^ For example, current clinical trials (NCT04115345, NCT04115345, and NCT00981006) uses a gelatin hydrogel with cells and/or growth factors. The hydrogels facilitate tissue healing following an injury (to the kidney or myocardium) by generating a suitable microenvironment, within which cell adhesion is paramount. Other common hydrogel materials, such as polyethylene glycol and polyacrylamide, are non‐adhesive and must be modified chemically with adhesive ligands to facilitate cell infiltration.^49^ However, for other applications discussed in this paper, such as marking tumor margins (e.g., TraceIT), rapid biodegradability is a design limitation, and therefore it is advantageous to use a hydrogel that degrades slowly over weeks to months.

Immune responses to injected biomaterials have been an active area of research for many years.^50^, ^51^ In seemingly all applied cases, it is important to minimize any immune response to the hydrogel injection. Immunological responses are responsible for adverse outcomes of biomaterial injection or implantation, including inflammation and fibrosis.^52^ While these responses are deleterious in their own right, their associated physicochemical shifts (i.e., changes in the local pH or temperature) can also alter the hydrogel material, impairing function further. Therefore, minimizing the host immune response to hydrogel injection is a critical biological design parameter.

### 5.4 Technological challenges

Despite the success of hydrogel‐based delivery systems, key technological challenges including chemistry, manufacturing and controls, defined regulatory guidelines, and practical adaptability remain as major roadblocks in their successful clinical translation. Since hydrogel fabrication is complex and varies between hydrogel systems, the development costs through clinical translation range in estimation from $50 million up to $800 million.^30^ In this section, we discuss the major translational barriers that must be addressed in order to improve healthcare with an inflating arsenal of injectable hydrogel‐based scaffolds and delivery systems.

### 5.5 Scale‐up strategies and GMP processes

A major hurdle in the clinical translation and integration of biomaterials‐based hydrogels is their compatibility with current good manufacturing practices (cGMPs). Since most hydrogel systems are synthesized in small batches at preclinical stage, efforts to translate fabrication/synthesis strategies to scaled systems are required. Batch variations, robustness, safety and efficiency issues are inevitable when performed at a larger scale. Natural polymer hydrogels may face additional difficulties, since natural polymers are heterogeneous and can exhibit different properties or characteristics at the molecular scale and potentially after synthesis into hydrogels. Additionally, the high‐water content of hydrogels makes the sterilization, storage and fabrication processes even more demanding.

### 5.6 Regulatory approvals

The diversity of crosslinking agents and biomaterials employed to develop hydrogel scaffolds, renders their regulatory classification and approval challenging. Unlike drugs which are broadly classified, hydrogels are classified under the “devices” category which according to the Section 201(g) of the FD&C Act covers “any product which does not achieve its primary intended purposes through chemical action within or on the body”. Furthermore, other than few exceptions, majority of the hydrogel‐based products are required to undergo additional FDA review of a 510(k) Pre‐Market Notification submission for obtaining legal marketing rights in the United States. In case of hydrogel scaffolds encompassing a drug or drug‐secreting cells, they are considered as a combination product, and thus their regulatory approval takes up to 7–10 years, which further limits their commercial viability.^30^

## 6 CONCLUSIONS

Injectable hydrogel systems provide a tunable platform to enable sustained release of small molecules or biologics and can also serve as a bulk material to interface between biological surface for application in tissue engineering or regeneration. Many examples of clinically and commercially successful injectable hydrogels exist **(**Table 1 **)** and the newer injectable hydrogel systems that are being investigated in the clinic **(**Table 2 **)** expand on these indications and likewise highlight the continued interest in development of these system. Still, injectable hydrogels face challenges unlike other therapeutic platforms, given their reliance on polymers, mechanical and solute transport properties, degradability, compatibility and scale‐up approaches. Altogether, the application of hydrogels toward improving healthcare remains a highly active area of research with a growing number of technologies being evaluated in humans.
